# 5,11-Ditosyl-5*H*,11*H*-dibenzo[*b*,*f*][1,5]diazo­cine-6,12-dione acetic acid hemisolvate

**DOI:** 10.1107/S1600536813007903

**Published:** 2013-03-28

**Authors:** Najat Abbassi, Oulemda Bassou, El Mostapha Rakib, Mohamed Saadi, Lahcen El Ammari

**Affiliations:** a Laboratoire de Chimie Organique et Analytique, Université Sultan Moulay Slimane, Faculté des Sciences et Techniques, Béni-Mellal, BP 523, Morocco; b Laboratoire de Chimie du Solide Appliquée, Faculté des Sciences, Université Mohammed V-Agdal, Avenue Ibn Battouta, BP 1014, Rabat, Morocco

## Abstract

The mol­ecular structure of the title compound, C_28_H_22_N_2_O_6_S_2_·0.5CH_3_COOH, is built up from three fused rings, two six and one eight membered. The eight-membered ring shows a boat conformation and the dihedral angle between the two benzene groups attached thereto is 66.43 (11)°, resulting in a V-shaped geometry. Two tosyl substituents are bound to the N atoms. The planes through the tolyl rings are roughly perpendicular, as indicated by the dihedral angle of 82.44 (12)°. In the crystal, the mol­ecule and its inversion-related symmetry-equivalent are linked to the acetic acid solvent mol­ecule by non-classical O—H⋯O and C—H⋯O hydrogen bonds. Two half-occupied acetic acid solvent mol­ecules are disordered at the same site and linked by a center of symmetry.

## Related literature

For the pharmacological activity of sulfonamides, see: Brzozowski *et al.* (2010 ▶); Drew (2000 ▶); Garaj *et al.* (2005 ▶). For their anti­proliferative activity, see: Abbassi *et al.* (2012 ▶); Bouissane *et al.* (2006 ▶); Lopez *et al.* (2010 ▶). For puckering parameters, see: Cremer & Pople (1975 ▶).
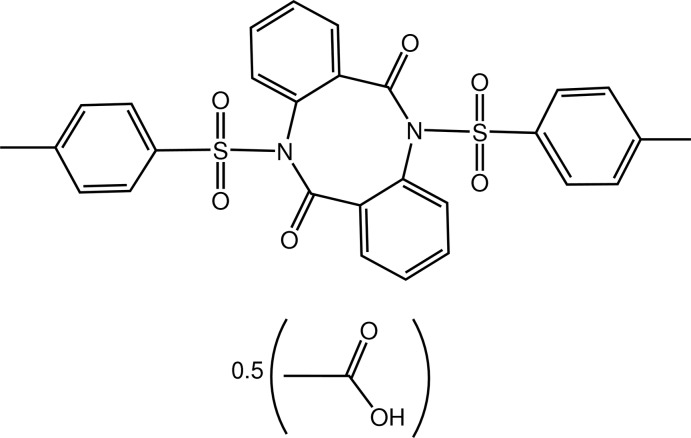


## Experimental

### 

#### Crystal data

C_28_H_22_N_2_O_6_S_2_·0.5C_2_H_4_O_2_
*M* *_r_* = 576.62
Triclinic, 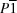

*a* = 8.6933 (11) Å
*b* = 11.1746 (18) Å
*c* = 14.8051 (19) Å
α = 87.042 (4)°
β = 74.370 (5)°
γ = 75.097 (4)°
*V* = 1338.2 (3) Å^3^
*Z* = 2
Mo *K*α radiation
μ = 0.25 mm^−1^
*T* = 296 K
0.41 × 0.35 × 0.27 mm

#### Data collection

Bruker X8 APEX diffractometer
19664 measured reflections
5446 independent reflections
4262 reflections with *I* > 2σ(*I*)
*R*_int_ = 0.039

#### Refinement

*R*[*F* ^2^ > 2σ(*F* ^2^)] = 0.042
*wR*(*F* ^2^) = 0.114
*S* = 1.02
5446 reflections
370 parameters
H-atom parameters constrained
Δρ_max_ = 0.41 e Å^−3^
Δρ_min_ = −0.47 e Å^−3^

### 

Data collection: *APEX2* (Bruker, 2009 ▶); cell refinement: *SAINT* (Bruker, 2009 ▶); data reduction: *SAINT*; program(s) used to solve structure: *SHELXS97* (Sheldrick, 2008 ▶); program(s) used to refine structure: *SHELXL97* (Sheldrick, 2008 ▶); molecular graphics: *ORTEP-3 for Windows* (Farrugia, 2012 ▶); software used to prepare material for publication: *PLATON* (Spek, 2009 ▶) and *publCIF* (Westrip, 2010 ▶).

## Supplementary Material

Crystal structure: contains datablock(s) global. DOI: 10.1107/S1600536813007903/im2423sup1.cif

Additional supplementary materials:  crystallographic information; 3D view; checkCIF report

## Figures and Tables

**Table 1:** Hydrogen-bond geometry (Å, °)

*D*—H⋯*A*	*D*—H	H⋯*A*	*D*⋯*A*	*D*—H⋯*A*
O8—H8⋯O3	0.95	2.66	3.460 (3)	142
C30—H30*C*⋯O3^i^	0.96	2.71	3.473 (5)	136
C16—H16⋯O2	0.93	2.49	3.190 (3)	132
C11—H11⋯O4^ii^	0.93	2.54	3.241 (2)	133

Symmetry codes: (i) 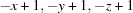; (ii) 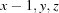.

**Fig. 1.**
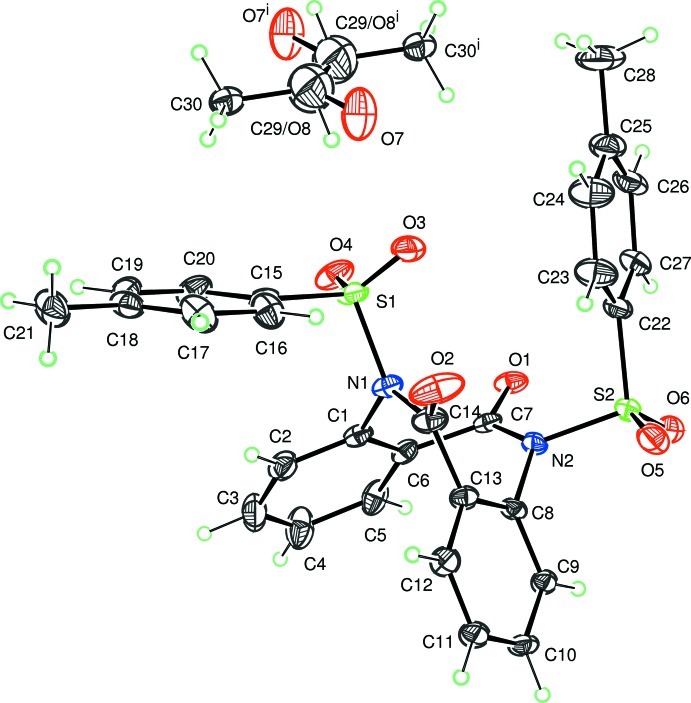
Molecular structure of the title compound with the atom-labelling scheme. Displacement ellipsoids are drawn at the 50% probability level. H atoms are represented as small circles.

## supplementary crystallographic information

### Comment

Sulfonamides constitute an important class of drugs. They possess various types of pharmacological activities such as antibacterial, hypoglycemic, anti-inflammatory, and antitumor (Lopez, *et al.*, 2010), as well as anti-carbonic anhydrase (Brzozowski, *et al.*, 2010), hypoglycemic (Drew, 2000), and anticancer activity (Garaj, *et al.*, 2005). The present work is part of research concerning the synthesis of some new N-(6 (4)-indazolyl)arylsulfonamide derivatives reported recently by our group. Some of these compounds showed an important antiproliferative activity against some human and murine cell lines (Abbassi, *et al.*, 2012, Bouissane *et al.*, 2006).

The three fused six- and eight-membered rings in the molecule of the title compound, are linked to two tolyl rings by sulfonyl groups as shown in Fig.1. The eight-membered ring displays a boat conformation, as indicated by the total puckering amplitude QT = 1.4807 (22) Å and spherical polar angles θ2 = 89.89 (8) and θ3 = 177 (3)° (Cremer & Pople, 1975). The dihedral angle between the two phenyl groups attached to the boat ring is 66.43 (11)°, resulting in a V shaped geometry. The planes through the two tolyl rings (C15 to C20) and (C22 to C27) are almost perpendicular as indicated by the dihedral angle between them of 82.44 (12)°.

In the crystal, each molecule and its symmetry through the inversion center are linked to the acetic acid solvent by O8—H8···O3, C30—H30c···O3^i^, C16—H16···O2 and C11—H11···O4^ii^ non-classical hydrogen bonds (Table 2).

Two half acetic acid solvent molecules are disordered at the same site of the crystal structure and linked by a center of symmetry.

### Experimental

A mixture of 2-nitrobenzaldehyde (1.22 mmol) and anhydrous SnCl_2_ (1.1 g, 6.1 mmol) in 25 mL of absolute ethanol was stirred for 1 h. After reduction, the starting material disappeared, and the solution was allowed to cool down. The pH was adjusted to 7–8 by addition of 5% aqueous potassium bicarbonate before extraction with ethyl acetate. The organic phase was washed with brine and dried over magnesium sulfate. The solvent was removed to afford the amine, which was immediately dissolved in pyridine (5 ml) and then reacted with 4-methylbenzenesulfonyl chloride (0.26 g, 1.25 mmol) at room temperature for 24 h. After the reaction mixture was concentrated *in vacuo*, the resulting residue was purified by flash chromatography (eluted with ethyl acetate : hexane 3:7). Colourless prisms of the title compound suitable for X-ray structure determination were collected after recrystallization from ethyl acetate : hexane (3:7 *v*/*v*) by slow evaporation of the solvent at room temperature after some days.

### Refinement

H atoms were located from a difference Fourier map and treated as riding with C–H = 0.96 and C–H = 0.93 Å for methyl and aromatic CH, respectively. Thermal parameters of hydrogen atoms were refined with *U*_iso_(H) = 1.2 *U*_eq_ for aromatic and *U*_iso_(H) = 1.5 *U*_eq_ for methyl hydrogen atoms. The refinement of the two half molecule acetic acid required the use of some constraints. Indeed, C29 and O8 occupy the same position with equal share and their atomic displacments are coupled. All sites of the atoms forming the acetic acid molecule are half filled except the one containing C29 and O8. The two half acetic acid molecule are linked by a center of symmetry.

### Crystal data

**Table:**

C_28_H_22_N_2_O_6_S_2_·0.5C_2_H_4_O_2_	*Z* = 2
*M**_r_* = 576.62	*F*(000) = 600
Triclinic, *P*1	*D*_x_ = 1.431 Mg m^−^^3^
Hall symbol: -p 1	Mo *K*α radiation, λ = 0.71073 Å
*a* = 8.6933 (11) Å	Cell parameters from 5446 reflections
*b* = 11.1746 (18) Å	θ = 2.4–26.4°
*c* = 14.8051 (19) Å	µ = 0.25 mm^−^^1^
α = 87.042 (4)°	*T* = 296 K
β = 74.370 (5)°	Prismatic, colourless
γ = 75.097 (4)°	0.41 × 0.35 × 0.27 mm
*V* = 1338.2 (3) Å^3^	

### Data collection

**Table:**

Bruker X8 APEX diffractometer	4262 reflections with *I* > 2σ(*I*)
Radiation source: fine-focus sealed tube	*R*_int_ = 0.039
Graphite monochromator	θ_max_ = 26.4°, θ_min_ = 2.4°
φ and ω scans	*h* = −10→8
19664 measured reflections	*k* = −13→13
5447 independent reflections	*l* = −18→18

### Refinement

**Table:**

Refinement on *F*^2^	Primary atom site location: structure-invariant direct methods
Least-squares matrix: full	Secondary atom site location: difference Fourier map
*R*[*F*^2^ > 2σ(*F*^2^)] = 0.042	Hydrogen site location: inferred from neighbouring sites
*wR*(*F*^2^) = 0.114	H-atom parameters constrained
*S* = 1.02	*w* = 1/[σ^2^(*F*_o_^2^) + (0.0555*P*)^2^ + 0.7987*P*] where *P* = (*F*_o_^2^ + 2*F*_c_^2^)/3
5446 reflections	(Δ/σ)_max_ = 0.001
370 parameters	Δρ_max_ = 0.41 e Å^−^^3^
0 restraints	Δρ_min_ = −0.47 e Å^−^^3^

### Special details

**Table:**

Geometry. All e.s.d.'s (except the e.s.d. in the dihedral angle between two l.s. planes) are estimated using the full covariance matrix. The cell e.s.d.'s are taken into account individually in the estimation of e.s.d.'s in distances, angles and torsion angles; correlations between e.s.d.'s in cell parameters are only used when they are defined by crystal symmetry. An approximate (isotropic) treatment of cell e.s.d.'s is used for estimating e.s.d.'s involving l.s. planes.
Refinement. Refinement of *F*^2^ against all reflections. The weighted *R*-factor *wR* and goodness of fit *S* are based on *F*^2^, conventional *R*-factors *R* are based on *F*, with *F* set to zero for negative *F*^2^. The threshold expression of *F*^2^ > σ(*F*^2^) is used only for calculating *R*-factors(gt) *etc*. and is not relevant to the choice of reflections for refinement. *R*-factors based on *F*^2^ are statistically about twice as large as those based on *F*, and *R*- factors based on all data will be even larger.

### Fractional atomic coordinates and isotropic or equivalent isotropic displacement parameters (Å^2^)

**Table:**

	*x*	*y*	*z*	*U*_iso_*/*U*_eq_	Occ. (<1)
C1	0.1314 (2)	0.04736 (19)	0.66644 (14)	0.0182 (4)	
C2	0.1389 (3)	−0.0156 (2)	0.58638 (15)	0.0243 (5)	
H2	0.1228	0.0280	0.5331	0.029*	
C3	0.1703 (3)	−0.1427 (2)	0.58609 (16)	0.0325 (5)	
H3	0.1734	−0.1850	0.5329	0.039*	
C4	0.1973 (3)	−0.2080 (2)	0.66490 (16)	0.0352 (6)	
H4	0.2197	−0.2940	0.6642	0.042*	
C5	0.1909 (3)	−0.1454 (2)	0.74484 (15)	0.0247 (5)	
H5	0.2103	−0.1896	0.7973	0.030*	
C6	0.1555 (2)	−0.01681 (19)	0.74673 (14)	0.0176 (4)	
C7	0.1413 (2)	0.04925 (19)	0.83511 (14)	0.0169 (4)	
C8	−0.1527 (2)	0.13023 (18)	0.83944 (13)	0.0152 (4)	
C9	−0.2611 (2)	0.06755 (18)	0.89469 (14)	0.0171 (4)	
H9	−0.2425	0.0310	0.9499	0.021*	
C10	−0.3982 (2)	0.06016 (19)	0.86638 (14)	0.0205 (4)	
H10	−0.4706	0.0166	0.9021	0.025*	
C11	−0.4279 (3)	0.1172 (2)	0.78530 (14)	0.0224 (5)	
H11	−0.5206	0.1123	0.7672	0.027*	
C12	−0.3208 (2)	0.1815 (2)	0.73125 (14)	0.0209 (4)	
H12	−0.3419	0.2204	0.6772	0.025*	
C13	−0.1806 (2)	0.18787 (18)	0.75783 (14)	0.0175 (4)	
C14	−0.0645 (3)	0.2544 (2)	0.69667 (15)	0.0232 (5)	
C15	0.2483 (3)	0.24962 (19)	0.48932 (14)	0.0194 (4)	
C16	0.1234 (3)	0.3396 (2)	0.46486 (16)	0.0274 (5)	
H16	0.0445	0.3938	0.5101	0.033*	
C17	0.1187 (3)	0.3471 (2)	0.37219 (17)	0.0303 (5)	
H17	0.0352	0.4066	0.3552	0.036*	
C18	0.2361 (3)	0.2677 (2)	0.30412 (15)	0.0250 (5)	
C19	0.3592 (3)	0.1785 (2)	0.33051 (16)	0.0278 (5)	
H19	0.4384	0.1247	0.2852	0.033*	
C20	0.3662 (3)	0.1682 (2)	0.42303 (15)	0.0268 (5)	
H20	0.4485	0.1078	0.4402	0.032*	
C21	0.2294 (3)	0.2763 (2)	0.20329 (17)	0.0356 (6)	
H21A	0.3194	0.2144	0.1662	0.053*	
H21B	0.2377	0.3570	0.1804	0.053*	
H21C	0.1267	0.2630	0.1991	0.053*	
C22	0.1091 (2)	0.31288 (19)	0.92998 (14)	0.0195 (4)	
C23	0.0855 (3)	0.4133 (2)	0.87229 (18)	0.0333 (6)	
H23	−0.0072	0.4346	0.8492	0.040*	
C24	0.2022 (3)	0.4814 (2)	0.8495 (2)	0.0395 (6)	
H24	0.1867	0.5494	0.8111	0.047*	
C25	0.3419 (3)	0.4508 (2)	0.88264 (18)	0.0324 (6)	
C26	0.3612 (3)	0.3508 (2)	0.94126 (17)	0.0292 (5)	
H26	0.4532	0.3301	0.9650	0.035*	
C27	0.2464 (3)	0.2812 (2)	0.96518 (15)	0.0239 (5)	
H27	0.2611	0.2139	1.0044	0.029*	
C28	0.4722 (3)	0.5229 (3)	0.8558 (2)	0.0508 (8)	
H28A	0.4406	0.5887	0.8150	0.076*	
H28B	0.5760	0.4687	0.8241	0.076*	
H28C	0.4827	0.5571	0.9113	0.076*	
O8	0.4362 (4)	0.5295 (3)	0.47416 (19)	0.0648 (8)	0.50
H8	0.3384	0.5054	0.5060	0.097*	0.50
C29	0.4362 (4)	0.5295 (3)	0.47416 (19)	0.0648 (8)	0.50
C30	0.4689 (5)	0.4823 (4)	0.3786 (3)	0.0253 (9)	0.50
H30A	0.3799	0.5254	0.3529	0.038*	0.50
H30B	0.4762	0.3951	0.3790	0.038*	0.50
H30C	0.5707	0.4971	0.3408	0.038*	0.50
O7	0.3037 (5)	0.5905 (3)	0.5167 (3)	0.0489 (10)	0.50
N1	0.0986 (2)	0.18137 (16)	0.66594 (12)	0.0186 (4)	
N2	−0.00829 (19)	0.13646 (15)	0.86839 (11)	0.0164 (4)	
O1	0.25131 (17)	0.03120 (13)	0.87404 (10)	0.0209 (3)	
O2	−0.1071 (2)	0.36032 (16)	0.67434 (14)	0.0436 (5)	
O3	0.2235 (2)	0.36337 (15)	0.64412 (11)	0.0315 (4)	
O4	0.40207 (18)	0.15004 (16)	0.61277 (11)	0.0303 (4)	
O5	−0.20005 (18)	0.30902 (15)	0.96957 (11)	0.0310 (4)	
O6	−0.0125 (2)	0.15277 (15)	1.04057 (10)	0.0287 (4)	
S1	0.25694 (6)	0.24198 (5)	0.60663 (4)	0.02218 (14)	
S2	−0.04108 (6)	0.22883 (5)	0.96340 (4)	0.02004 (14)	

### Atomic displacement parameters (Å^2^)

**Table:**

	*U*^11^	*U*^22^	*U*^33^	*U*^12^	*U*^13^	*U*^23^
C1	0.0126 (10)	0.0221 (11)	0.0204 (10)	−0.0069 (8)	−0.0035 (8)	0.0043 (8)
C2	0.0239 (11)	0.0320 (12)	0.0178 (10)	−0.0102 (10)	−0.0045 (9)	0.0051 (9)
C3	0.0425 (14)	0.0328 (13)	0.0190 (11)	−0.0085 (11)	−0.0029 (10)	−0.0051 (9)
C4	0.0491 (16)	0.0214 (12)	0.0251 (12)	−0.0001 (11)	−0.0009 (11)	−0.0022 (9)
C5	0.0255 (12)	0.0243 (12)	0.0178 (10)	0.0008 (9)	−0.0021 (9)	0.0038 (9)
C6	0.0110 (9)	0.0225 (11)	0.0175 (10)	−0.0033 (8)	−0.0022 (8)	0.0030 (8)
C7	0.0134 (10)	0.0202 (10)	0.0186 (10)	−0.0086 (8)	−0.0038 (8)	0.0069 (8)
C8	0.0105 (9)	0.0169 (10)	0.0200 (10)	−0.0036 (8)	−0.0066 (8)	−0.0015 (8)
C9	0.0145 (10)	0.0211 (10)	0.0168 (10)	−0.0056 (8)	−0.0051 (8)	0.0009 (8)
C10	0.0139 (10)	0.0264 (11)	0.0235 (11)	−0.0101 (9)	−0.0042 (8)	0.0016 (9)
C11	0.0160 (10)	0.0329 (12)	0.0224 (11)	−0.0096 (9)	−0.0085 (8)	0.0006 (9)
C12	0.0176 (10)	0.0272 (12)	0.0196 (10)	−0.0046 (9)	−0.0093 (8)	0.0036 (8)
C13	0.0132 (10)	0.0168 (10)	0.0223 (10)	−0.0036 (8)	−0.0049 (8)	0.0013 (8)
C14	0.0197 (11)	0.0251 (12)	0.0279 (11)	−0.0085 (9)	−0.0097 (9)	0.0078 (9)
C15	0.0216 (11)	0.0240 (11)	0.0187 (10)	−0.0144 (9)	−0.0092 (8)	0.0095 (8)
C16	0.0365 (13)	0.0183 (11)	0.0285 (12)	−0.0044 (10)	−0.0131 (10)	0.0013 (9)
C17	0.0431 (14)	0.0190 (11)	0.0345 (13)	−0.0049 (10)	−0.0237 (11)	0.0059 (9)
C18	0.0340 (13)	0.0258 (12)	0.0243 (11)	−0.0173 (10)	−0.0149 (10)	0.0079 (9)
C19	0.0229 (11)	0.0371 (13)	0.0230 (11)	−0.0092 (10)	−0.0043 (9)	0.0027 (10)
C20	0.0164 (11)	0.0371 (13)	0.0271 (12)	−0.0072 (10)	−0.0075 (9)	0.0094 (10)
C21	0.0499 (16)	0.0386 (14)	0.0274 (12)	−0.0171 (12)	−0.0204 (11)	0.0054 (10)
C22	0.0189 (10)	0.0189 (10)	0.0235 (10)	−0.0088 (8)	−0.0062 (8)	−0.0018 (8)
C23	0.0315 (13)	0.0292 (13)	0.0463 (15)	−0.0118 (11)	−0.0201 (11)	0.0097 (11)
C24	0.0443 (16)	0.0269 (13)	0.0523 (16)	−0.0172 (12)	−0.0155 (13)	0.0126 (12)
C25	0.0271 (13)	0.0231 (12)	0.0455 (15)	−0.0120 (10)	−0.0002 (11)	−0.0082 (10)
C26	0.0215 (11)	0.0292 (12)	0.0418 (14)	−0.0104 (10)	−0.0111 (10)	−0.0081 (10)
C27	0.0258 (12)	0.0239 (11)	0.0278 (11)	−0.0102 (9)	−0.0127 (9)	−0.0005 (9)
C28	0.0350 (15)	0.0342 (15)	0.081 (2)	−0.0223 (13)	0.0026 (14)	−0.0080 (14)
O8	0.0622 (18)	0.081 (2)	0.0552 (17)	−0.0210 (16)	−0.0189 (14)	−0.0014 (15)
C29	0.0622 (18)	0.081 (2)	0.0552 (17)	−0.0210 (16)	−0.0189 (14)	−0.0014 (15)
C30	0.020 (2)	0.024 (2)	0.031 (2)	−0.0067 (18)	−0.0053 (18)	0.0017 (18)
O7	0.042 (2)	0.036 (2)	0.048 (2)	0.0165 (18)	0.0000 (18)	−0.0187 (18)
N1	0.0158 (9)	0.0224 (9)	0.0197 (9)	−0.0085 (7)	−0.0056 (7)	0.0066 (7)
N2	0.0119 (8)	0.0215 (9)	0.0184 (8)	−0.0061 (7)	−0.0063 (6)	−0.0018 (7)
O1	0.0144 (7)	0.0274 (8)	0.0258 (8)	−0.0099 (6)	−0.0107 (6)	0.0090 (6)
O2	0.0271 (9)	0.0274 (10)	0.0717 (13)	−0.0078 (7)	−0.0091 (9)	0.0254 (9)
O3	0.0396 (10)	0.0400 (10)	0.0240 (8)	−0.0280 (8)	−0.0070 (7)	0.0044 (7)
O4	0.0172 (8)	0.0515 (11)	0.0263 (8)	−0.0142 (7)	−0.0102 (6)	0.0159 (7)
O5	0.0176 (8)	0.0341 (9)	0.0403 (10)	−0.0046 (7)	−0.0046 (7)	−0.0164 (7)
O6	0.0370 (9)	0.0392 (9)	0.0173 (8)	−0.0229 (8)	−0.0071 (7)	0.0027 (7)
S1	0.0199 (3)	0.0336 (3)	0.0191 (3)	−0.0163 (2)	−0.0078 (2)	0.0087 (2)
S2	0.0173 (3)	0.0259 (3)	0.0197 (3)	−0.0102 (2)	−0.0041 (2)	−0.0039 (2)

### Geometric parameters (Å, º)

**Table:**

C1—C2	1.387 (3)	C18—C21	1.507 (3)
C1—C6	1.393 (3)	C19—C20	1.385 (3)
C1—N1	1.451 (3)	C19—H19	0.9300
C2—C3	1.376 (3)	C20—H20	0.9300
C2—H2	0.9300	C21—H21A	0.9600
C3—C4	1.386 (3)	C21—H21B	0.9600
C3—H3	0.9300	C21—H21C	0.9600
C4—C5	1.387 (3)	C22—C23	1.381 (3)
C4—H4	0.9300	C22—C27	1.386 (3)
C5—C6	1.390 (3)	C22—S2	1.751 (2)
C5—H5	0.9300	C23—C24	1.382 (3)
C6—C7	1.496 (3)	C23—H23	0.9300
C7—O1	1.214 (2)	C24—C25	1.386 (4)
C7—N2	1.392 (3)	C24—H24	0.9300
C8—C9	1.383 (3)	C25—C26	1.382 (3)
C8—C13	1.390 (3)	C25—C28	1.511 (3)
C8—N2	1.450 (2)	C26—C27	1.382 (3)
C9—C10	1.388 (3)	C26—H26	0.9300
C9—H9	0.9300	C27—H27	0.9300
C10—C11	1.385 (3)	C28—H28A	0.9600
C10—H10	0.9300	C28—H28B	0.9600
C11—C12	1.380 (3)	C28—H28C	0.9600
C11—H11	0.9300	O8—O7	1.199 (4)
C12—C13	1.397 (3)	O8—C30	1.465 (5)
C12—H12	0.9300	O8—O8^i^	1.510 (5)
C13—C14	1.488 (3)	O8—H8	0.9518
C14—O2	1.203 (3)	C30—H30A	0.9600
C14—N1	1.407 (3)	C30—H30B	0.9600
C15—C20	1.382 (3)	C30—H30C	0.9600
C15—C16	1.391 (3)	O7—H8	0.9283
C15—S1	1.755 (2)	N1—S1	1.7000 (17)
C16—C17	1.381 (3)	N2—S2	1.7052 (17)
C16—H16	0.9300	O3—S1	1.4215 (17)
C17—C18	1.384 (3)	O4—S1	1.4287 (16)
C17—H17	0.9300	O5—S2	1.4229 (16)
C18—C19	1.389 (3)	O6—S2	1.4260 (16)
			
C2—C1—C6	120.69 (19)	C18—C21—H21B	109.5
C2—C1—N1	119.37 (18)	H21A—C21—H21B	109.5
C6—C1—N1	119.95 (18)	C18—C21—H21C	109.5
C3—C2—C1	119.8 (2)	H21A—C21—H21C	109.5
C3—C2—H2	120.1	H21B—C21—H21C	109.5
C1—C2—H2	120.1	C23—C22—C27	120.87 (19)
C2—C3—C4	120.2 (2)	C23—C22—S2	119.55 (16)
C2—C3—H3	119.9	C27—C22—S2	119.52 (16)
C4—C3—H3	119.9	C22—C23—C24	118.8 (2)
C3—C4—C5	120.1 (2)	C22—C23—H23	120.6
C3—C4—H4	119.9	C24—C23—H23	120.6
C5—C4—H4	119.9	C23—C24—C25	121.6 (2)
C4—C5—C6	120.2 (2)	C23—C24—H24	119.2
C4—C5—H5	119.9	C25—C24—H24	119.2
C6—C5—H5	119.9	C26—C25—C24	118.4 (2)
C5—C6—C1	118.95 (19)	C26—C25—C28	119.8 (2)
C5—C6—C7	119.38 (17)	C24—C25—C28	121.8 (2)
C1—C6—C7	121.67 (18)	C27—C26—C25	121.2 (2)
O1—C7—N2	122.21 (19)	C27—C26—H26	119.4
O1—C7—C6	123.48 (18)	C25—C26—H26	119.4
N2—C7—C6	114.29 (16)	C26—C27—C22	119.2 (2)
C9—C8—C13	121.32 (17)	C26—C27—H27	120.4
C9—C8—N2	118.90 (17)	C22—C27—H27	120.4
C13—C8—N2	119.78 (17)	C25—C28—H28A	109.5
C8—C9—C10	118.93 (18)	C25—C28—H28B	109.5
C8—C9—H9	120.5	H28A—C28—H28B	109.5
C10—C9—H9	120.5	C25—C28—H28C	109.5
C11—C10—C9	120.46 (19)	H28A—C28—H28C	109.5
C11—C10—H10	119.8	H28B—C28—H28C	109.5
C9—C10—H10	119.8	O7—O8—C30	122.8 (3)
C12—C11—C10	120.37 (18)	O7—O8—O8^i^	119.8 (4)
C12—C11—H11	119.8	C30—O8—O8^i^	115.6 (3)
C10—C11—H11	119.8	O7—O8—H8	49.5
C11—C12—C13	119.90 (18)	C30—O8—H8	102.6
C11—C12—H12	120.0	O8^i^—O8—H8	106.1
C13—C12—H12	120.0	O8—C30—H30A	108.4
C8—C13—C12	119.01 (18)	O8—C30—H30B	110.4
C8—C13—C14	122.23 (17)	H30A—C30—H30B	109.5
C12—C13—C14	118.75 (18)	O8—C30—H30C	109.6
O2—C14—N1	123.2 (2)	H30A—C30—H30C	109.5
O2—C14—C13	123.0 (2)	H30B—C30—H30C	109.5
N1—C14—C13	113.85 (17)	O8—O7—H8	51.3
C20—C15—C16	121.33 (19)	C14—N1—C1	119.97 (16)
C20—C15—S1	119.85 (16)	C14—N1—S1	122.16 (14)
C16—C15—S1	118.81 (17)	C1—N1—S1	116.64 (13)
C17—C16—C15	118.7 (2)	C7—N2—C8	120.46 (16)
C17—C16—H16	120.6	C7—N2—S2	120.92 (13)
C15—C16—H16	120.6	C8—N2—S2	116.82 (13)
C16—C17—C18	121.2 (2)	O3—S1—O4	120.61 (10)
C16—C17—H17	119.4	O3—S1—N1	106.68 (9)
C18—C17—H17	119.4	O4—S1—N1	104.34 (9)
C17—C18—C19	118.8 (2)	O3—S1—C15	110.00 (10)
C17—C18—C21	120.8 (2)	O4—S1—C15	108.42 (10)
C19—C18—C21	120.4 (2)	N1—S1—C15	105.68 (9)
C20—C19—C18	121.2 (2)	O5—S2—O6	119.90 (10)
C20—C19—H19	119.4	O5—S2—N2	103.20 (8)
C18—C19—H19	119.4	O6—S2—N2	109.03 (9)
C15—C20—C19	118.7 (2)	O5—S2—C22	109.77 (10)
C15—C20—H20	120.7	O6—S2—C22	109.15 (10)
C19—C20—H20	120.7	N2—S2—C22	104.62 (9)
C18—C21—H21A	109.5		
			
C6—C1—C2—C3	0.1 (3)	C24—C25—C26—C27	1.2 (4)
N1—C1—C2—C3	179.86 (19)	C28—C25—C26—C27	−178.4 (2)
C1—C2—C3—C4	−1.2 (4)	C25—C26—C27—C22	−0.4 (3)
C2—C3—C4—C5	0.7 (4)	C23—C22—C27—C26	−0.5 (3)
C3—C4—C5—C6	0.8 (4)	S2—C22—C27—C26	−177.60 (17)
C4—C5—C6—C1	−1.8 (3)	O2—C14—N1—C1	−158.1 (2)
C4—C5—C6—C7	177.1 (2)	C13—C14—N1—C1	20.7 (3)
C2—C1—C6—C5	1.4 (3)	O2—C14—N1—S1	8.8 (3)
N1—C1—C6—C5	−178.39 (18)	C13—C14—N1—S1	−172.40 (14)
C2—C1—C6—C7	−177.50 (18)	C2—C1—N1—C14	92.5 (2)
N1—C1—C6—C7	2.7 (3)	C6—C1—N1—C14	−87.8 (2)
C5—C6—C7—O1	57.8 (3)	C2—C1—N1—S1	−75.2 (2)
C1—C6—C7—O1	−123.3 (2)	C6—C1—N1—S1	104.62 (18)
C5—C6—C7—N2	−123.7 (2)	O1—C7—N2—C8	−160.90 (18)
C1—C6—C7—N2	55.2 (2)	C6—C7—N2—C8	20.6 (2)
C13—C8—C9—C10	1.5 (3)	O1—C7—N2—S2	3.3 (3)
N2—C8—C9—C10	−179.16 (17)	C6—C7—N2—S2	−175.14 (13)
C8—C9—C10—C11	−1.6 (3)	C9—C8—N2—C7	93.3 (2)
C9—C10—C11—C12	0.5 (3)	C13—C8—N2—C7	−87.3 (2)
C10—C11—C12—C13	0.7 (3)	C9—C8—N2—S2	−71.5 (2)
C9—C8—C13—C12	−0.3 (3)	C13—C8—N2—S2	107.86 (18)
N2—C8—C13—C12	−179.68 (18)	C14—N1—S1—O3	34.98 (18)
C9—C8—C13—C14	−179.19 (19)	C1—N1—S1—O3	−157.71 (14)
N2—C8—C13—C14	1.5 (3)	C14—N1—S1—O4	163.68 (16)
C11—C12—C13—C8	−0.8 (3)	C1—N1—S1—O4	−29.01 (16)
C11—C12—C13—C14	178.13 (19)	C14—N1—S1—C15	−82.08 (18)
C8—C13—C14—O2	−125.5 (2)	C1—N1—S1—C15	85.23 (15)
C12—C13—C14—O2	55.7 (3)	C20—C15—S1—O3	137.50 (17)
C8—C13—C14—N1	55.7 (3)	C16—C15—S1—O3	−41.45 (19)
C12—C13—C14—N1	−123.1 (2)	C20—C15—S1—O4	3.7 (2)
C20—C15—C16—C17	−0.2 (3)	C16—C15—S1—O4	−175.27 (16)
S1—C15—C16—C17	178.73 (17)	C20—C15—S1—N1	−107.71 (18)
C15—C16—C17—C18	−0.5 (3)	C16—C15—S1—N1	73.34 (18)
C16—C17—C18—C19	0.6 (3)	C7—N2—S2—O5	175.75 (15)
C16—C17—C18—C21	179.7 (2)	C8—N2—S2—O5	−19.48 (16)
C17—C18—C19—C20	−0.1 (3)	C7—N2—S2—O6	−55.75 (17)
C21—C18—C19—C20	−179.2 (2)	C8—N2—S2—O6	109.02 (15)
C16—C15—C20—C19	0.7 (3)	C7—N2—S2—C22	60.89 (17)
S1—C15—C20—C19	−178.24 (17)	C8—N2—S2—C22	−134.34 (15)
C18—C19—C20—C15	−0.5 (3)	C23—C22—S2—O5	−31.5 (2)
C27—C22—C23—C24	0.5 (4)	C27—C22—S2—O5	145.58 (17)
S2—C22—C23—C24	177.5 (2)	C23—C22—S2—O6	−164.81 (18)
C22—C23—C24—C25	0.4 (4)	C27—C22—S2—O6	12.3 (2)
C23—C24—C25—C26	−1.3 (4)	C23—C22—S2—N2	78.6 (2)
C23—C24—C25—C28	178.3 (3)	C27—C22—S2—N2	−104.27 (18)

Symmetry code: (i) −*x*+1, −*y*+1, −*z*+1.

### Hydrogen-bond geometry (Å, º)

**Table:**

*D*—H···*A*	*D*—H	H···*A*	*D*···*A*	*D*—H···*A*
O8—H8···O3	0.95	2.66	3.460 (3)	142
C30—H30*C*···O3^i^	0.96	2.71	3.473 (5)	136
C16—H16···O2	0.93	2.49	3.190 (3)	132
C11—H11···O4^ii^	0.93	2.54	3.241 (2)	133

Symmetry codes: (i) −*x*+1, −*y*+1, −*z*+1; (ii) *x*−1, *y*, *z*.

## References

[bb1] Abbassi, N., Chicha, H., Rakib, E. M., Hannioui, A., Alaoui, M., Hajjaji, A., Geffken, D., Aiello, C., Gangemi, R., Rosano, C. & Viale, M. (2012). *Eur. J. Med. Chem.* **57**, 240–249. 10.1016/j.ejmech.2012.09.013 23072738

[bb2] Bouissane, L., El Kazzouli, S., Léonce, S., Pffeifer, P., Rakib, E. M., Khouili, M. & Guillaumet, G. (2006). *Bioorg. Med. Chem.* **14**, 1078–1088. 10.1016/j.bmc.2005.09.037 16274996

[bb3] Bruker (2009). *APEX2*, *SAINT* and *SADABS* Bruker AXS Inc., Madison, Wisconsin, USA.

[bb4] Brzozowski, Z., S1awiński, J., Saczewski, F., Innocenti, A., Supuran, C. T. (2010). *Eur. J. Med. Chem.* **45**, 2396–2404. 10.1016/j.ejmech.2010.02.020 20202722

[bb5] Cremer, D. & Pople, J. A. (1975). *J. Am. Chem. Soc.* **97**, 1354–1358.

[bb6] Drew, J. (2000). *Science*, **287**, 1960–964.

[bb7] Farrugia, L. J. (2012). *J. Appl. Cryst.* **45**, 849–854.

[bb8] Garaj, V., Puccetti, L., Fasolis, G., Winum, J. Y., Montero, J. L., Scozzafava, A., Vullo, D., Innocenti, A. & Supuran, C. T. (2005). *Bioorg. Med. Chem. Lett.* **15**, 3102–3108. 10.1016/j.bmcl.2005.04.056 15905091

[bb9] Lopez, M., Bornaghi, L. F., Innocenti, A., Vullo, D., Charman, S. A., Supuran, C. T. & Poulsen, S.-A. (2010). *J. Med. Chem.* **53**, 2913–2926. 10.1021/jm901888x 20201556

[bb10] Sheldrick, G. M. (2008). *Acta Cryst.* A**64**, 112–122. 10.1107/S0108767307043930 18156677

[bb11] Spek, A. L. (2009). *Acta Cryst.* D**65**, 148–155. 10.1107/S090744490804362X PMC2631630 19171970

[bb12] Westrip, S. P. (2010). *J. Appl. Cryst.* **43**, 920–925.

